# Gastric juice microbiota in pediatric chronic gastritis that clinically tested positive and negative for *Helicobacter pylori*

**DOI:** 10.3389/fmicb.2023.1112709

**Published:** 2023-04-25

**Authors:** Ying Chen, Shou-Yue Xia, Fu-Xia Ru, Jun-Jie Feng, Ji Tao, Zhi-Yuan Wei, Xiu Li, Chengjia Qian, Qiong Lin, Jian-Huan Chen

**Affiliations:** ^1^Department of Gastroenterology, Affiliated Children’s Hospital of Jiangnan University, Wuxi, China; ^2^Laboratory of Genomic and Precision Medicine, Wuxi School of Medicine, Jiangnan University, Wuxi, Jiangsu, China; ^3^Laboratory Animal Center, Jiangnan University, Wuxi, Jiangsu, China; ^4^Department of General Surgery, Affiliated Hospital of Jiangnan University, Wuxi, China

**Keywords:** gastric microbiota, *Helicobacter pylori*, pediatric chronic gastritis, network of microbial interaction, *Streptococcus*

## Abstract

**Purpose:**

*Helicobacter pylori* (HP) infection is an identified risk factor for pediatric chronic gastritis (PCG), but its impact on gastric juice microbiota (GJM) remains to be further elucidated in PCG. This study aimed to analyze and compare the microbial communities and microbial interactive networks of GJM in PCG that clinically tested positive and negative for HP (HP+ and HP−, respectively).

**Methods:**

A total of 45 PCG patients aged from 6 to 16 years were recruited, including 20 HP+ and 25 HP− patients tested by culture and rapid urease test. Gastric juice samples were collected from these PCG patients and subjected to high-throughput amplicon sequencing and subsequent analysis of 16S rRNA genes.

**Results:**

While no significant change in alpha diversity, significant differences in beta diversity were observed between HP+ and HP− PCG. At the genus level, *Streptococcus, Helicobacter*, and *Granulicatella* were significantly enriched in HP+ PCG, whereas *Campylobacter* and *Absconditabacteriales (SR1)* were significantly enriched in *HP*− PCG. Network analysis showed that *Streptococcus* was the only genus positively correlated with *Helicobacter* (*r* = 0.497) in the GJM net*work* of overall PCG. Moreover, compared to HP− PCG, HP+ PCG showed a reduction in microbial network connectivity in GJM. Netshift analysis identified driver microbes including *Streptococcus* and other four genera, which substantially contributed to the GJM network transition from HP− PCG to HP+ PCG. Furthermore, Predicted GJM function analysis indicated up-regulated pathways related to the metabolism of nucleotides, carbohydrates, and L-Lysine, the urea cycle, as well as endotoxin peptidoglycan biosynthesis and maturation in HP+ PCG.

**Conclusion:**

GJM in HP+ PCG exhibited dramatically altered beta diversity, taxonomic structure, and function, with reduced microbial network connectivity, which could be involved in the disease etiology.

## Introduction

Chronic gastritis is a common health problem in children (Sipponen and Maaroos, 2015). *Helicobacter pylori* (HP) infection is an important cause of chronic gastritis, which occurs generally in childhood (Sabbagh et al., 2019), and is associated with peptic ulcer, gastric atrophy, intestinal metaplasia, and gastric cancer. It is important to study the impact of HP for the understanding of the etiology of pediatric chronic gastritis (PCG).

HP plays a pivotal role in the gastric microbiota. The stomach is a special area in the digestive tract, with gastric acid secretion constituting its unique ecological environment and characteristic microbial community (Caguazango, 2020). HP could promote inflammation in the gastric mucosa, and leads to changes in the gastric mucosa microbiota (GMM) in PCG (Zheng et al., 2021). In addition, the gastric juice microbiota (GJM) is typically affected by various factors such as diet (Martinsen et al., 2019), which could result in greater variation compared to GMM. The diversity of GJM has been reported to be higher than that of GMM. Sung et al. found that the relative abundance of *Actinobacteria*, *Bacteroidetes,* and *Firmicutes* was relatively higher in GJM than in GMM (Sung et al., 2016). Besides, FLéJOU et al. found that non-HP bacteria could have explicit effects on gastritis (Fléjou et al., 1990), and *Streptococcus* showed a higher relative abundance in HP− cancer patients. (Sohn et al., 2017). It is also noteworthy that the collection of gastric juice is less invasive compared with gastric mucosal biopsy.

In the current study, we recruited a cohort of PCG and analyzed the impact of HP abundance on the microbial communities and co-occurrence networks of GJM.

## Materials and methods

### Study design and participants

This study was approved by the Medical Ethics Committee of Jiangnan University and conducted in accordance with the Declaration of Helsinki. A total of 45 PCG were recruited in the current study, including 27 boys and 18 girls, with an average age of 9.03 ± 0.93 years old. Informed consent was obtained from all of the participants after explanation of the nature of the study. Gastritis diagnosis was made based on endoscopic observation and pathological features of gastric atrophy. Exclusion criteria included treatment with antibiotics or proton pump inhibitors (PPIs) within 6 months; serious mental or organ damage; or poor patient compliance. HP infection status of these PCG patients was tested using gastric mucosal biopsy and rapid urease tests, and confirmed with HP culture on brain heart infusion agar plate supplemented with blood, inoculated with gastric mucosal biopsy tissue homogenate in a microaerophilic environment (85% N_2_, 10% CO_2_, 5% O_2_) at 37°C for 3 days.

### Sample collection and 16S rRNA gene sequencing

Gastric juice samples were collected from all participants with sterilized 1.5-mL tubes containing ethanol on ice, and stored at −80°C until analysis. The V4 hypervariable region of the 16S rRNA gene was amplified from the gastric fluid samples with V4-specific primers 515F (5′-GTGCCAGCMGCCGCGGTAA-3′) and 806R (5′-GGACTACHVGGGTWTCTAAT-3′). Amplicons were checked using the 2% agarose gel, and purified using GeneJET Gel Extraction Kit (Thermo Fisher Scientific, Waltham). Constructed libraries were sequenced on an Ion S5XL sequencer (Thermo Fisher Scientific, Waltham) with a single-end 400-bp read length configuration following standard protocols provided by the manufacturer.

### 16S rRNA gene sequence analysis

The 16S rRNA gene sequencing reads were analyzed using the QIIME2 (version 2020.11.0) analysis pipeline as previously described (Bolyen et al., 2019; Wei et al., 2022). In brief, low-quality and chimeric sequences were filtered using DADA2. Amplicon sequence variant (ASV) tables at 100% sequence similarity were generated. Taxonomy classification was assigned to ASVs using q2-feature-classifier and the SILVA database (release r132) at a 99% similarity cutoff (Glockner et al., 2017). Microbiota diversity was analyzed using QIIME2: alpha diversity metrics including Pielou’s evenness, the Chao1, Shannon and Simpson’s indices, and beta diversity including weighted/unweighted UniFrac distances, and Bray-Curtis distances followed by non-metric multidimensional scaling (NMDS) analysis and Principal Coordinate Analysis (PCoA). The linear discriminant analysis (LDA) effect size (LEfSe) algorithm was used to identify group-enriched taxa (Segata et al., 2011). Phylogenetic cladograms were drawn using GraPhlAn (version 1.1.3). PICRUSt2 was used to predict microbiota function based on the 16S rRNA sequencing data (Douglas et al., 2020).

### Microbial interactive network analysis

ASVs with average relative abundance >0.1% of the microbiome were subjected to correlation analysis of their occurrence patterns (Barberan et al., 2012; Cardinale et al., 2015). The SparCC algorithm was used to estimate the correlations among gut microbes(Friedman and Alm, 2012). 1,000 bootstrap replicates were applied to calculate the pseudo *p*-values, and correlations with |correlation coefficient |(*r*)| > 0.2 and *p* < 0.01 were considered significant. For each genus with significant SparCC correlations, its degree was calculated as an indicator of its weight in the network by summing up its edges. The SparCC network was further constructed using Cytoscape 3.9.1 (Shannon et al., 2003). In addition, NetShift ^2^ was used to evaluate potential driver microbes as described (Kuntal et al., 2019), and to calculate Neighbor shift (NESH) scores to quantify increased interactions in the network shift.

## Results

### Demographic and clinical characteristics of study subjects

The demographic and clinical characteristics of 45 PGC patients were summarized in Table 1. Endoscopic findings showed a significantly higher proportion of gastritis with additional clinical conditions (GAC) including duodenitis, bile reflux, duodenal ulcer, and gastric ulcer in HP+ PCG than in HP− PCG (OR = 4.97, 95% CI: 0.97–34.69, *p* = 0.040). No significant difference was found in age, gender, and other clinical features between HP+ and HP− PCG (all *p* > 0.05).

**Table 1:** Demographic and clinical characteristics of HP*−* and HP+ PCG in the current study.

Item	HP− (*n*, %)	HP+ (*n*, %)	*p* *
Age	9.92 ± 2.33	10.25 ± 2.61	0.317
Gender			1
Male	15 (60.00)	12 (60.00)	
Female	10 (40.00)	8 (40.00)	
Endoscopic finding			0.040^**^
Normal	2 (8.00)	0 (0.00)	–
Superficial gastritis and duodenitis	2 (8.00)	2 (10.00)	–
Superficial gastritis and bile reflux	0 (0.00)	3 (15.00)	–
Superficial gastritis and duodenal ulcer	0 (0.00)	2 (10.00)	–
Superficial gastritis and gastric ulcer	1 (4.00)	2 (10.00)	–
Superficial gastritis without other findings listed above	20 (80.00)	11 (55.00)	–
clinical symptoms			
Abdominal pain			0.465
No	6 (24.00)	5 (25.00)	–
Yes, 0–1 month	7 (28.00)	3 (15.00)	–
Yes, 1–3 months	2 (8.00)	4 (20.00)	–
Yes, 3–6 months	2 (8.00)	4 (20.00)	–
Yes, 6 months and above	8 (32.00)	4 (20.00)	–
Abdominal distension	3 (12.00)	1 (5.00)	0.617
Nausea	1 (4.00)	1 (5.00)	1.000
Belching	2 (8.00)	0 (0.00)	0.495
Ozostomia	2 (8.00)	3 (15.00)	0.642
Acid reflux	1 (4.00)	0 (0.00)	1.000

* *p*-values are calculated using Student’s *t* -test for continuous data or Fisher’s exact test for frequency data.

** The *p*-value is calculated by comparing the proportion of superficial gastritis with duodenitis, bile reflux, duodenal ulcer, or gastric ulcer in HP+ PCG and HP− PCG.

### GJM diversity in HP+ and HP− PCG

**Figure 1:**
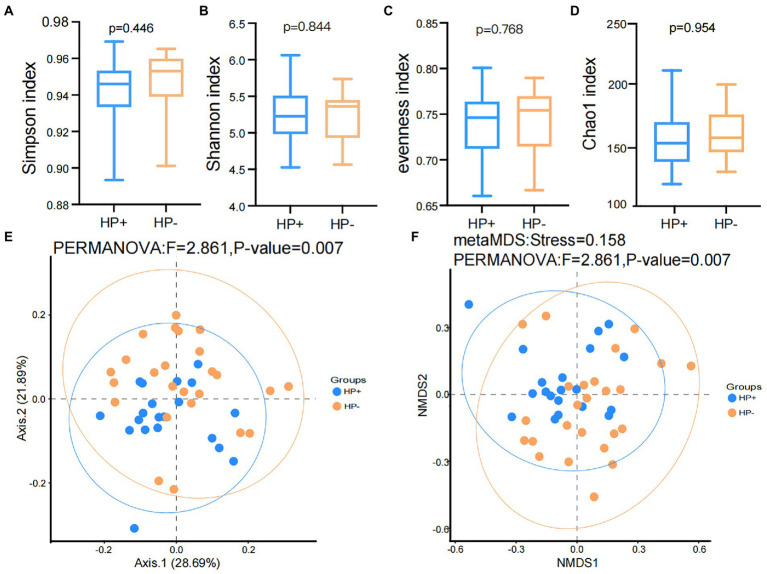
Comparison of Alpha and beta diversities between HP− and HP+ PCG. **(A)** Simpson index. **(B)** Shannon index. **(C)** Eveness index. **(D)** Chao1 index. **(E)** PCoA based on Bray-Curtis distances. **(F)** NMDS on Bray-Curtis distances.

The results of the rarefaction curve shown in Supplementary Figure S1 indicated that our current study’s sequencing data could sufficiently capture the majority of the microbial taxa. In addition, alpha diversity results showed no significant difference (all *p* > 0.05) in the Shannon, Simpson, evenness, and Chao1 (Figures 1A–D) indices between the two groups. Nevertheless, PCoA and NMDS results based on Bray-Curtis distances suggested a significant difference in beta diversity between HP+ and HP− PCG (PERMANOVA *p* = 0.007 and 0.007 respectively), although the two groups might not be completely separated (Figures 1E,F).

### GJM composition in HP+ and HP − PCG

**Figure 2:**
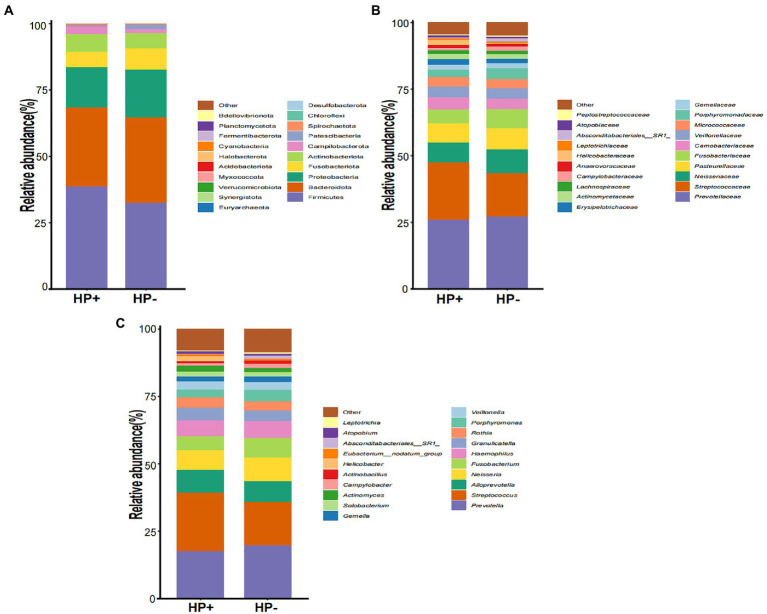
The microbial abundance of GJM in HP− and HP+ PCG. Microbial abundance is shown at the **(A)** phylum level, **(B)** family level, **(C)** and the genus level.

**Figure 3:**
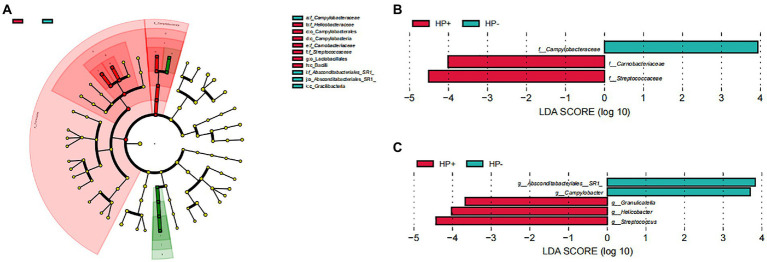
LEfSe analysis of GJM in HP− and HP+ PCG. Phylogenetic cladograms **(A)**, and barplots at **(B)** the family, and **(C)** Genus levels are shown.

GJM in both HP+ PCG and HP− PCG patients was dominated by 8 phyla, including Firmicutes, Bacteroidetes, Proteobacteria, Fusobacteriota, Actinobacteriota, Campilobacterota, Patescibacteria, and Spirochaetota (Figure 2A). *Prevotellaceae*, *Streptococcaceae*, *Neisseriaceae*, *Pasteurellaceae*, *Fusobacteriaceae*, *Carnobacteriaceae*, *Veillonellaceae, Micrococcaceae*, *Porphyromonadaceae*, and *Gemellaceae* were the top 10 families, and *Prevotella*, *Streptococcus*, *Alloprevotella*, *Neisseria*, *Fusobacterium*, *Haemophilus*, *Granulicatella*, *Rothia*, *Porphyromonas*, and *Veillonella* were the top 10 genera in PCG GJM (Figures 2B,C). LEfSe analysis was then applied to identify the most relevant taxa responsible for differences between the two groups. As shown in the cladogram in Figure 3A, at the phylum level, Firmicutes and Campilobacterota were enriched in *HP+* PCG. At the family level, *Streptococcaceae, Helicobacteraceae*, and *Carnobacteriaceae* were enriched in *HP+* PCG, whereas *Campylobacteraceae* and *Absconditabacteriales (SR1)* were enriched in *HP−* PCG (Figure 3B). At the genus level, *Streptococcus, Helicobacter*, and *Granulicatella* were enriched in HP+ PCG, whereas *Campylobacter* and *Absconditabacteriales (SR1)* were enriched in *HP−* PCG (Figure 3C and Supplementary Figure S2).

**Figure 4:**
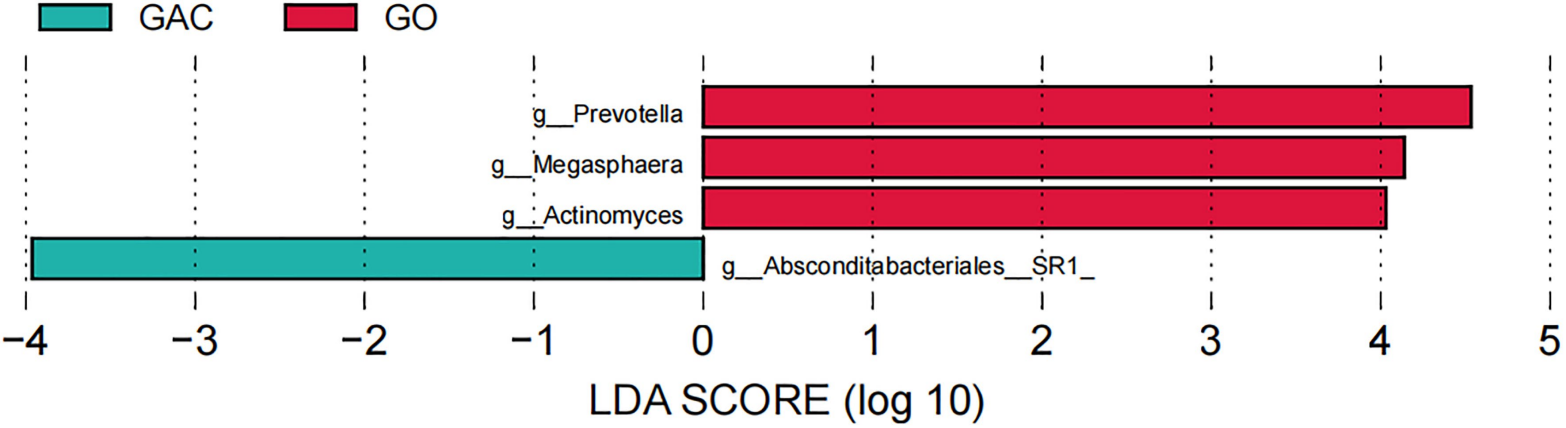
LEfSe analysis of GJM in GO and GAC subgroups of HP+ PCG.

In addition, as GAC was dramatically increased in HP*+ PCG,* we further divided *the* HP+ PCG patients into a group of gastritis only (GO) and a group of GAC according to the endoscopic findings, and compared GJM between the two groups. Our results showed that the genus *Absconditabacteriales (SR1)* was enriched in GO, whereas genera including *Prevotella*, *Megashaera*, and *Actinomyces* were enriched in GAC (Figure 4).

### GJM network in HP+ and HP− PCG

**Figure 5:**
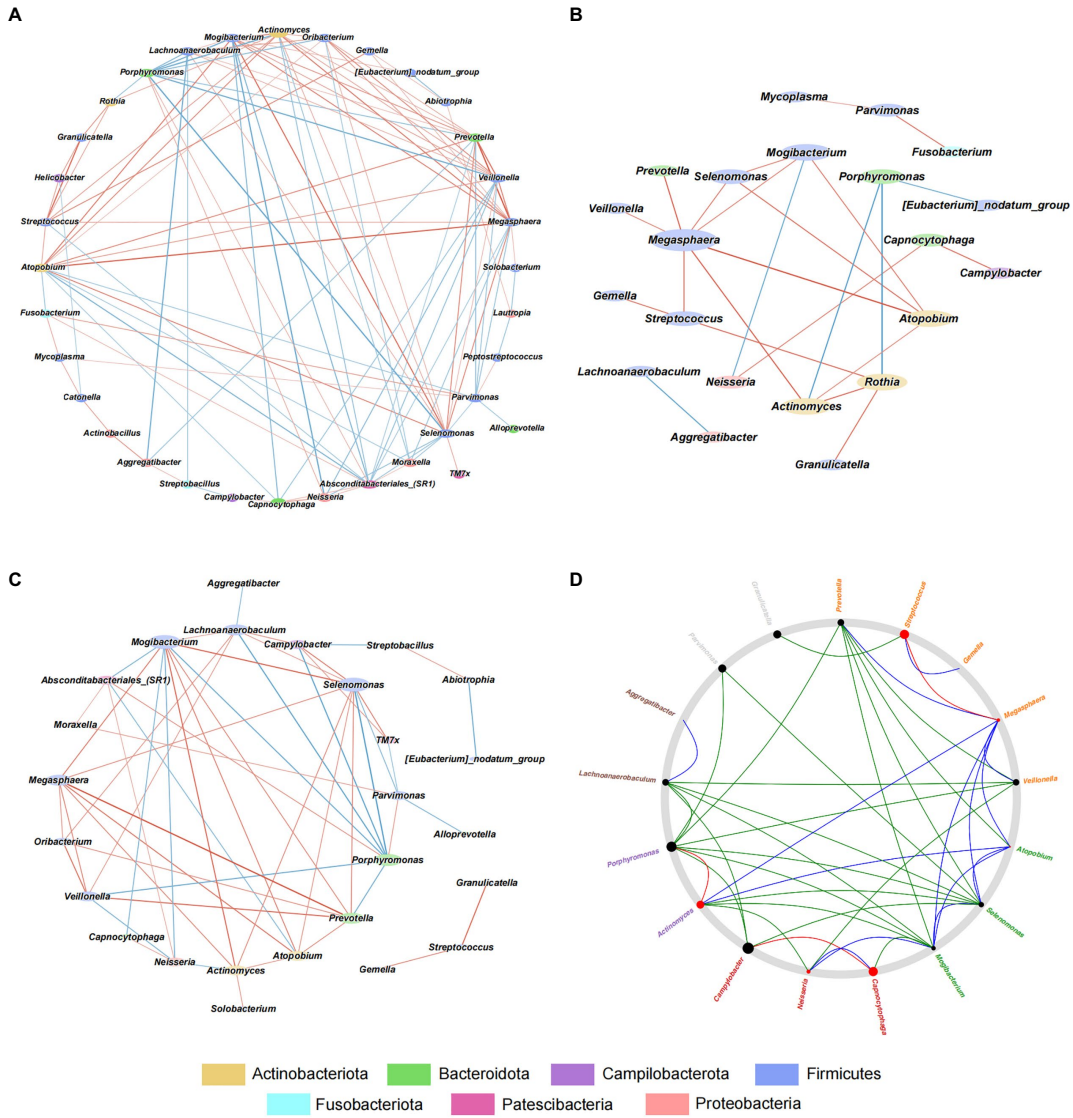
Interactive Network in HP−, HP+, and overall PCG at the genus level. **(A)** Interactive network analysis of HP− PCG. **(B)** Interactive network analysis of overall PCG. **(C)** Interactive Network analysis of HP+ PCG. **(D)** Netshift analysis of GJM between HP− and HP+ PCG.

To explore the interaction among microbes in GJM, we conducted a correlation analysis using the SparCC algorithm and the genus abundance of genera with average relative abundance ≥ 0.1%, and constructed microbial co-occurrence networks with cutoff set as |*r*| > = 0.2 and *p* <  0.01 in overall PCG, HP− PCG, and HP+ PCG. In the GJM network of overall PCG, *Streptococcus*, the most enriched genus in HP+ PCG, was found to be the only genus positively correlated with *Helicobacter* (*r* = 0.497) (Figure 5A). It was also positively correlated with *Granulicatella*, *Gemella*, *Actinomyces*, *Rothia*, *Atopobium*, and *Megasphaera*, (*r* = 0.746, 0.598, 0.504, 0.497, 0.484, 0.483, and 0.465, respectively), and negatively correlated with *Absconditabacteriales (SR1)*, (*r* = −0.402). No significant correlation between other microbes and *Helicobacter* was found in GJM networks of either HP+ PCG or HP− PCG. Notably, lower network connectivity was found in HP+ PCG compared to that in HP− PCG or overall PCG (Figures 5B,C, and Supplementary Tables S1–3). Similarly, *Actinomyces* and *Prevotella 6* were the dominant members in overall PCG (Figure 5B). In *HP+* PCG, *Streptococcus* showed positive correlations with *Rothia* (*r* = 0.716), *Megasphaera* (*r* = 0.708), and *Gemella* (*r* = 0.679), (Figure 5C). In *HP−* PCG, *Streptococcus* showed positive correlations with *Granulicatella* (*r* = 0.783) and *Gemella* (*r* = 0.621). Furthermore, *Streptococcus* was identified as a driver microbe responsible for the microbial changes between *HP+* and *HP−* PCG in Netshift analysis (Table 2, Figure 5D). Additional driver microbes identified by Netshift analysis included *Capnocytophaga*, *Actinomyces*, *Neisseria*, and *Megasphaera*.

**Table 2:** Genera with the top five NESH score in Netshift analysis.

Genus	n(HP−)	n(HP+)	core (HP+)	Intersect	NESH-score
*Campylobacter*	3	1	1	0	1.393
*Porphyromonas*	7	1	1	0	1.268
***Streptococcus***	2	2	1	1	1.143
*Capnocytophaga*	2	2	1	1	1.143
*Actinomyces*	5	3	2	2	0.976

### Predicted GJM function in HP+ and HP− PCG

**Figure 6:**
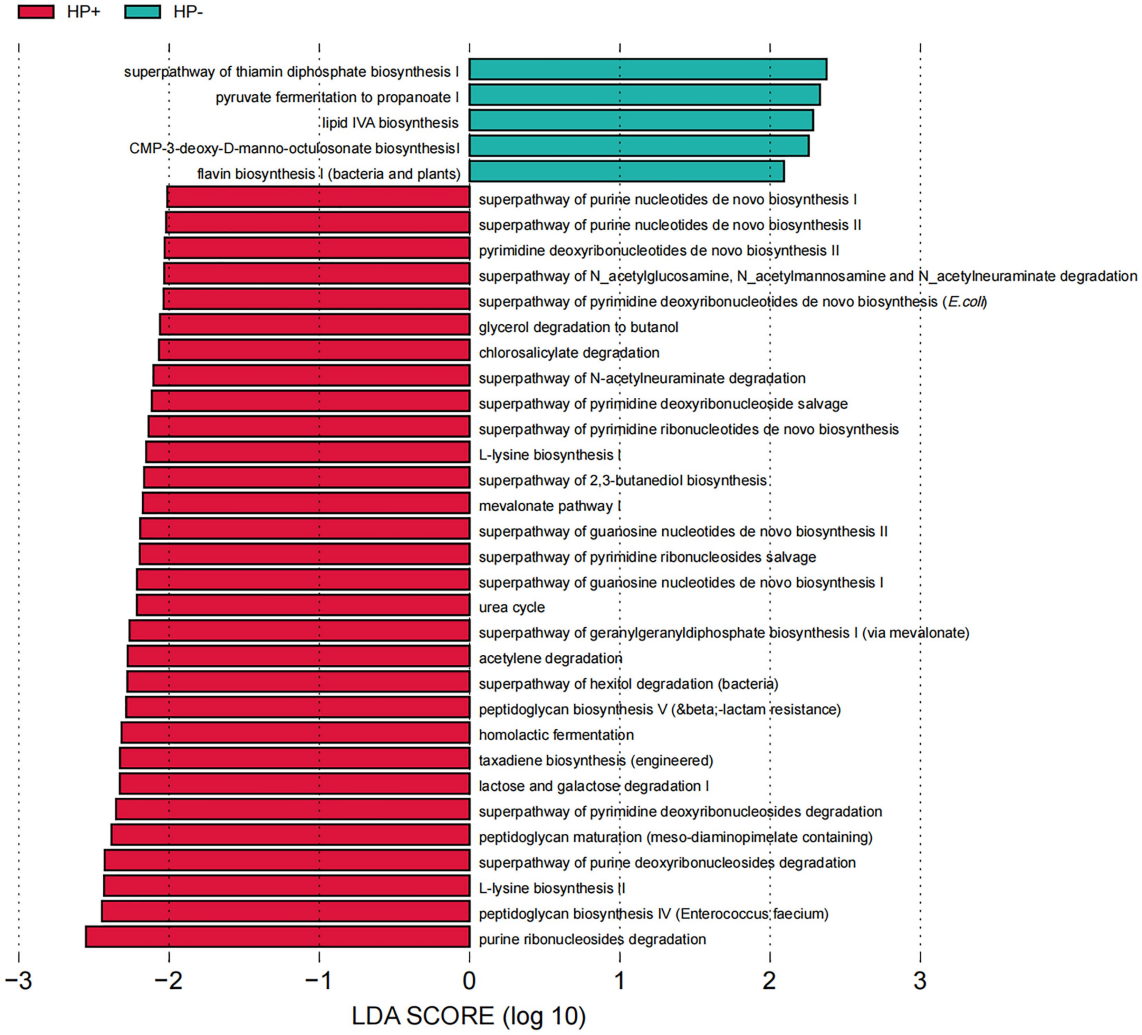
LEfSe analysis of GJM KEGG pathways differentially enriched in HP− and HP+ PCG.

PICRUSt2 predicted a dramatical difference in GJM function, with a total of 35 Kyoto Encyclopedia of Genes and Genomes (KEGG) pathways differentially abundant between HP+ and HP− PCG (Figure 6). The 30 pathways significantly upregulated in HP+ PCG were mainly related to the metabolism of nucleotides, carbohydrates, and L-Lysine, as well as endotoxin peptidoglycan biosynthesis and maturation. In addition, the urea cycle was also significantly upregulated in HP+ PCG. In contrast, pathways enriched in HP− PCG were related to the biosynthesis of thiamin diphosphate, CMP-3-deoxy-D-manno-octulosonat, flavin, and lipid IVA which was an intermediate in the biosynthetic pathway of lipid A and lipopolysaccharide (LPS).

## Discussions

Since Warren and Marshall first cultivated *HP* from children’s gastric mucosa, after two decades of in-depth research, *HP* has been identified as a pivotal pathogen of chronic gastritis and peptic ulcer (Kishikawa et al., 2020). More, the influence of HP on the gastric microbiota might contribute to the development of PCG. By analyzing GJM in HP+ and HP− PCG clinically tested by routine methods, our current study showed a substantial impact of HP abundance on GJM composition, microbial interactive network, and function.

By far, most existing studies have focused on the influence of HP on the gastrointestinal microbiota in adults (He et al., 2019; Tao et al., 2020), and there have been only limited studies in children, especially for GJM. Brawner et al. reported that HP infection significantly reduced the alpha diversity of GMM in PCG. Different from such results in GMM, our current study showed no significant difference in alpha diversity of GJM between HP+ and HP− PCG, pointing to different effects of HP on GMM and GJM. Notably, *Helicobacter* could also be detected in HP−PCG clinically tested *via* routine methods, yet with an extremely low relative abundance than in HP+ PCG. 16S amplicon sequencing was recently reported with higher sensitivity in detecting HP in the gastric microbiota, by revealing HP existence in some cases that were tested negative for HP *via* routine methods (Gantuya et al., 2021). *Helicobacter* is commonly found in human children and non-human primate infants (Wei et al., 2022). Such findings suggested that an extremely low abundance of HP might still exist in samples that test negative by routine clinical methods.

Our results showed a significant difference in beta diversity with dramatic changes in taxonomic structure between HP+ and HP*−* PCG. The higher abundance of Firmicutes in HP+ PCG was similar to the gut microbiota of asymptomatic children with HP infection (Benavides-Ward et al., 2018). In addition, our study showed that a non-HP bacterium *Streptococcus*, with a significantly positive correlation with *Helicobacter* in all PCG, was the most enriched genus in *HP+* PCG. *Streptococcus* is a gram-positive bacterium of spherical shape and has been reported to be involved in several diseases and health issues, such as gastritis (Minalyan et al., 2017). Moreover, our GJM networks revealed that *Streptococcus* acted as a driver that contributed to the shift of the GJM network from HP− PCG to HP+ PCG. Another non-HP bacterium *Granulicatella* is associated with atrophic gastritis or intestinal metaplasia in patients following successful HP eradication (Liu et al., 2022). In contrast, *Campylobacter* and *Absconditabacteriales (SR1)* were enriched in our *HP−* PCG patients. *Campylobacter* infection usually causes diarrhea, fever, and stomach cramps. *Absconditabacteriales* exhibited significantly lower abundance in the gastric microbiota in HP− Crohn’s disease patients than in control subjects (Ostrowski et al., 2021). Such findings indicated non-HP pathogens could play a potential role in PCG.

The change of non-*HP* pathogens in the stomach may be due to the change in pH value in gastric juice. Rosen et al. reported that acid suppression resulted in the overgrowth of gastric bacteria such as *Staphylococcus* and *Streptococcus* (Rosen et al., 2014). In addition, *Streptococcus*, *Actinomyces*, *Megasphaera*, and *Granulicatella* were significantly increased in patients receiving proton pump inhibitor treatment (Jackson et al., 2016; Takagi et al., 2018).

Recent studies have demonstrated the importance of microbiota networks in understanding microbiota changes in diseases and aging. The reduced network connectivity observed in HP+ PCG suggested a dramatic change in microbial interaction in the group compared to that of HP− PCG, emphasizing the impact of HP on the microbial community. In addition, *Streptococcus* showed a significant positive correlation with *Helicobacter* in overall PCG, and contributed to the network shift from HP− to HP+ PCG by interconnecting with multiple hub or driver microbes. Such findings thus highlighted the importance of non-HP pathogens.

Although no significant difference in clinical symptoms such as abdominal pain and belching between the two groups, endoscopic findings showed a significantly higher proportion of PCG with duodenitis, bile reflux, duodenal ulcer, or gastric ulcer. Such findings were in line with previous reports that HP was a cause of duodenitis (Ohkusa et al., 2003), peptic ulcer, and possibly bile reflux (Ladas et al., 1996). Furthermore, our results also indicated GJM might also contribute to the development of duodenitis, bile reflux, and peptic ulcer.

Gastric acid secretion in the stomach constitutes a characteristic microbial community and its function in the gastric juice (Caguazango, 2020), which could be involved in HP infection and the development of PCG. Our PICRUSt2 results implicated a dramatic difference in the GJM function between HP+ and HP− PCG. In line with the positive findings in the clinical rapid urease test, the urea cycle pathway could be significantly upregulated in HP+ PCG compared to HP− PCG. In addition, our findings also indicated that biosynthesis and maturation of PGN were also enhanced in HP+ PCG compared to HP− PCG. PGN is a major cell wall component of Gram-positive bacteria. It is reported that HP cag + strains deliver components of PGN into epithelial cells *via* the cag secretion system, leading to decreased apoptosis, increased proliferation, and increased cell migration (Suarez et al., 2017). These changes indicated that PGN could play a potentially important role in modulating host inflammatory responses to HP, allowing the bacteria to persist and induce carcinogenic consequences in the gastric niche (Suarez et al., 2017). Interestingly, we also found that L-lysine biosynthesis increased significantly in HP+ PCG. L-lysine could significantly delay and inhibit gastric emptying (Uchida et al., 2017), which might be related to symptoms such as abdominal distension caused by HP.

Due to sample limitations, further studies will be necessary to confirm our findings in other cohorts with larger sample sizes. Nevertheless, the results of our current study demonstrate dramatic differences in GJM between HP+ and HP− PCG in terms of taxonomic structure, microbiota network, and function. GJM analysis could be a less invasive way to monitor and study the gastric microbiota dynamics in PCG.

## Data availability statement

The raw sequence data presented in the study are deposited in the Genome Sequence Archive in the National Genomics Data Center, China National Center for Bioinformation/Beijing Institute of Genomics, Chinese Academy of Sciences (GSA-human), accession number PRJCA014620.

## Ethics statement

This study was approved by the Ethics Committee of Jiangnan University, and was conducted in accordance with the Declaration of Helsinki. Written informed consent to participate in this study was provided by the participants’ legal guardian/next of kin.

## Author contributions

YC, S-YX, F-XR, and J-JF performed the data analysis and wrote the manuscript. YC collected the clinical data. JT and Z-YW conducted the experiments. XL and CQ contributed to the discussion. QL and J-HC conceived the project and planned the experiments. All authors contributed to the final manuscript.

## Funding

This study was supported in part by grants from the Youth Research Program of Wuxi Health Commission (Q202011), Wuxi maternal and child health research project (FYKY202108), and Wuxi Medical Innovation Team (CXTD2021011).

## Conflict of interest

The authors declare that the research was conducted in the absence of any commercial or financial relationships that could be construed as a potential conflict of interest.

## Publisher’s note

All claims expressed in this article are solely those of the authors and do not necessarily represent those of their affiliated organizations, or those of the publisher, the editors and the reviewers. Any product that may be evaluated in this article, or claim that may be made by its manufacturer, is not guaranteed or endorsed by the publisher.

## Footnotes

^2^ https://web.rniapps.net/netshift/
