# Supplementary material for: Gastric juice microbiota in pediatric chronic gastritis that clinically tested positive and negative for Helicobacter pylori
Source: Front Microbiol. 2023 Apr 25;14:1112709. doi: 10.3389/fmicb.2023.1112709 (PMC10168005; doi:10.3389/fmicb.2023.1112709)
Supplement: Supplementary file 1 [file Data_Sheet_1.docx]

**
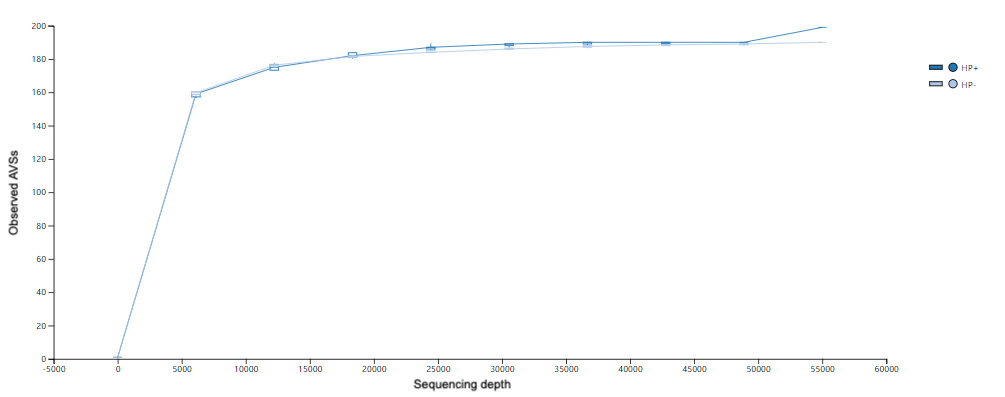
**

**Supplementary Figure S1. Rarefaction analysis of observed ASVs in PCG**

**
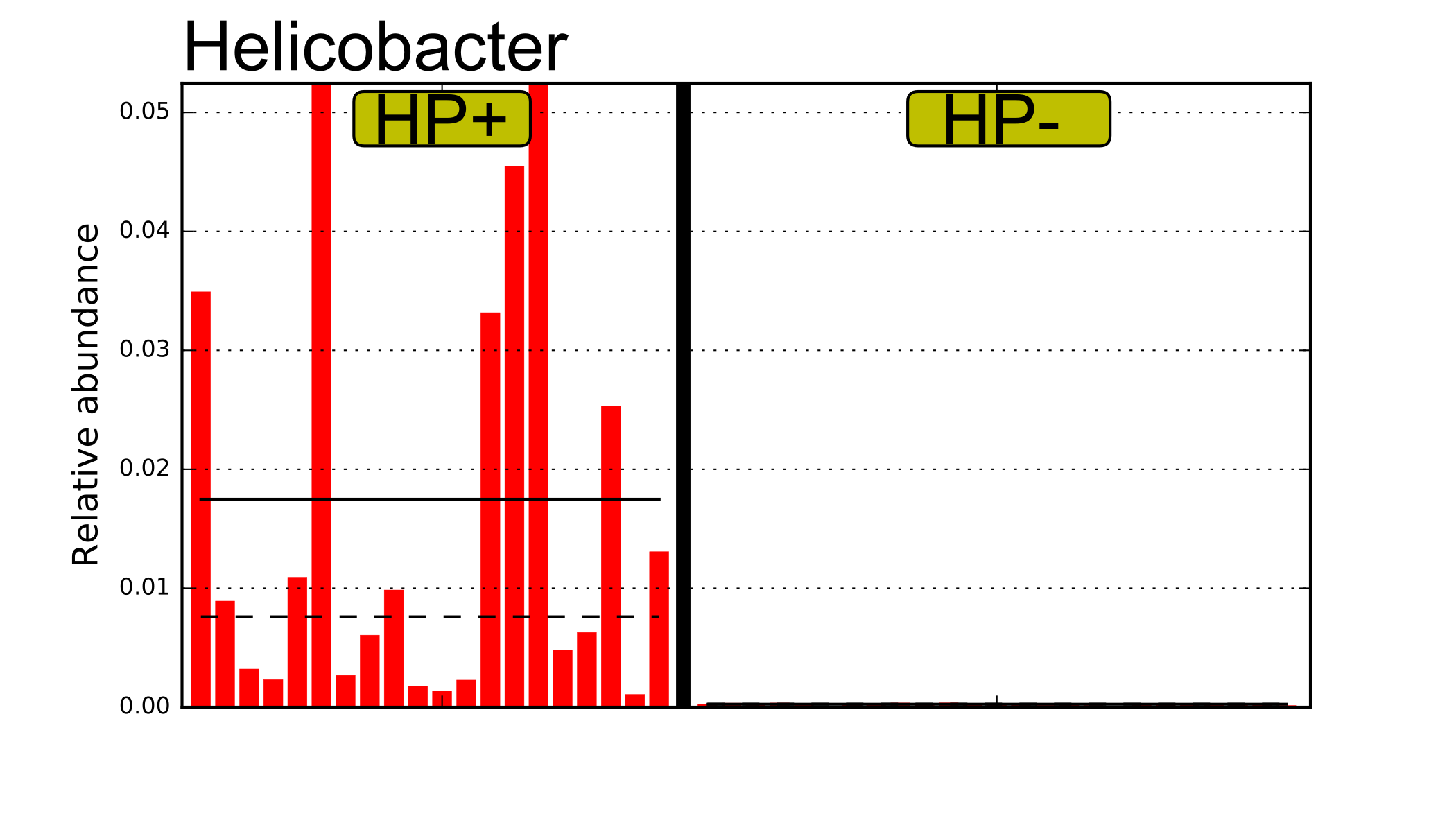
**

**Supplementary Figure S2. Relative abundance of Helicobacter in individual samples**
